# The gut-liver axis in animal diseases: roles, mechanisms, and veterinary clinical applications

**DOI:** 10.3389/fvets.2026.1812064

**Published:** 2026-04-01

**Authors:** Fuwan Peng, Shijie Tang, Yutong Cui, Yuyou Zeng

**Affiliations:** 1 College of Bioscience and Biotechnology, Hunan Agricultural University, Changsha, China; 2 College of Veterinary Medicine, Hunan Agricultural University, Changsha, China

**Keywords:** gut microbiota, gut-liver axis, livestock, mechanisms, poultry

## Abstract

The liver serves as the core organ for metabolism, detoxification, and immune regulation in animals. Its functional homeostasis directly determines the animal's health status and production performance. The gut-liver axis, as a critical inter-organ regulatory network connecting the intestine and the liver, plays a vital role in regulating immune responses, maintaining nutritional metabolism balance, and preventing pathogen invasion. The review systematically elucidates the central role of the gut-liver axis in regulating metabolic homeostasis and immune defense in animals. It comprehensively integrates the structural foundations, regulatory mechanisms, and pathological functions of this axis across ruminants, pigs and poultry. It focuses on disruptions to intestinal barrier integrity and dynamic alterations in the gut microbiota and its metabolites (such as short-chain fatty acids, bile acids, etc.). These alterations directly or indirectly influence hepatic metabolism, immunity, detoxification functions and systemic inflammation, thereby contributing to the pathogenesis of various liver and related metabolic disorders. It provides intervention strategies based on gut-liver axis regulation, such as dietary interventions, probiotics/prebiotics, and microbiota-directed modulation. It also explores the application of artificial intelligence (AI) and big data modeling in monitoring the gut-liver axis with the goal of developing early warning systems. By integrating multi-omics technologies to identify key regulatory factors specific to the gut-liver axis, this work extends beyond the conventional gut-centric, single-organ framework to encompass multi-organ synergistic interactions. This approach provides novel theoretical insights and technical support for the discovery of multi-target bioactive compounds and the advancement of precision disease prevention strategies.

## 1 Introduction

In livestock production, liver health is central to ensuring growth performance, immune stability, and overall animal wellbeing. As a metabolic center, the integrity of liver function is directly linked to production efficiency. The liver not only serves as the nexus for energy and nutrient metabolism, participating in the transformation and transport of nutritional molecules—including the generation, breakdown, modification, and storage of glucose/glycogen, proteins, and lipids, as well as coordinating the storage, metabolism, and activation of certain vitamins and the storage of minerals—but also plays a critical role in fat digestion and absorption through the secretion of bile (1, 2). Simultaneously, it acts as the most vital detoxification center, reducing the toxicity of drugs and endotoxins through conjugation, oxidation, reduction, and hydrolysis, and even breaking them down into non-toxic compounds excreted in urine or bile, thereby effectively clearing endogenous and exogenous toxins such as mycotoxins, drug residues, and metabolic waste (3, 4). Furthermore, the liver's robust immune function cannot be overlooked. Its resident Kupffer cell phagocytic system efficiently clears pathogens from the bloodstream (5, 6), and it serves as a key synthesis site for various immunoglobulins (IgG, IgA, IgM) and complement proteins (C3, C4), playing a pivotal role in the animal's non-specific immunity (6, 7).

Although the liver of different livestock and poultry is responsible for core functions such as metabolism, detoxification, and bile secretion, there are differences in its physiological functions. The core function of the liver of ruminants (such as cattle and sheep) is to convert a large amount of volatile fatty acids (VFAs), mainly including acetic acid, propionic acid and butyric acid, produced by microorganisms fermenting fibrous substances in the rumen, into energy forms that the body can directly utilize for the needs of key physiological processes such as lactation (8, 9). Furthermore, research has revealed the central role of the liver in nutrient allocation in ruminants. Changes in dietary structure (such as the ratio of concentrate to roughage) directly alter the liver's amino acid metabolism patterns. Additionally, the liver's ability to synthesize urea is closely related to amino acid metabolism. Through the urea cycle, it detoxifies large amounts of ammonia produced in the rumen, and through the urea recycling mechanism, it conserves nitrogen sources to adapt to low-protein diets (10–12). Unlike mammals, poultry fat tissue does not produce new fat, and more than 70% of the fatty acids are synthesized in the liver (13). To adapt the high egg production rate and rapid growth demands, the liver needs to continuously synthesize large amounts of very low-density lipoproteins (VLDL) to transport triglycerides to adipose tissue and ovaries, which leads to the high susceptibility of poultry to fatty liver hemorrhagic syndrome (FLHS) in high-energy feeding models (14, 15).

In modern intensive farming, strategies commonly adopted to pursue high-efficiency production—such as high-energy diets and excessive feeding—along with the potential presence of mycotoxins, heavy metal contamination, and the misuse of antibiotics in feed, collectively impose a burden on the liver (16–18). For example, high energy diet in pregnant cows triggers energy deficit leading to fat mobilization, liver triglyceride accumulation leads to fatty liver, milk yield decrease, and early elimination risk increases by 50% (19, 20). FLHS is not only the leading cause of non-communicable death but also has a mortality rate as high as 74% during the peak egg-laying period in caged hens, while egg production decreases by 5%−30% (21, 22). Additionally, chronic liver damage caused by mycotoxins can reduce the feed conversion rate (FCR) of meat and poultry by 5%−20%, significantly increasing production costs (23, 24). However, the liver does not function as the core hub of the body's metabolism through an independent system. More and more researches suggest a close causal relationship between gut microbiome imbalance and the occurrence and development of liver diseases (25, 26). The gut-liver axis, as a key bridge connecting the gut microenvironment to liver function, has recently become a hot topic in deciphering the pathogenesis of liver diseases. Most previous studies have primarily observed the physiological and pathological mechanisms of the “gut-liver axis” in a single species, with insufficient attention paid to species-specific differences. Therefore, this paper will systematically elaborate on the regulatory mechanisms, pathological effects, and potential intervention targets of the gut-liver axis in various animal models, providing a theoretical basis for the prevention and treatment of animal liver diseases.

## 2 The gut-liver axis

**Figure 1:**
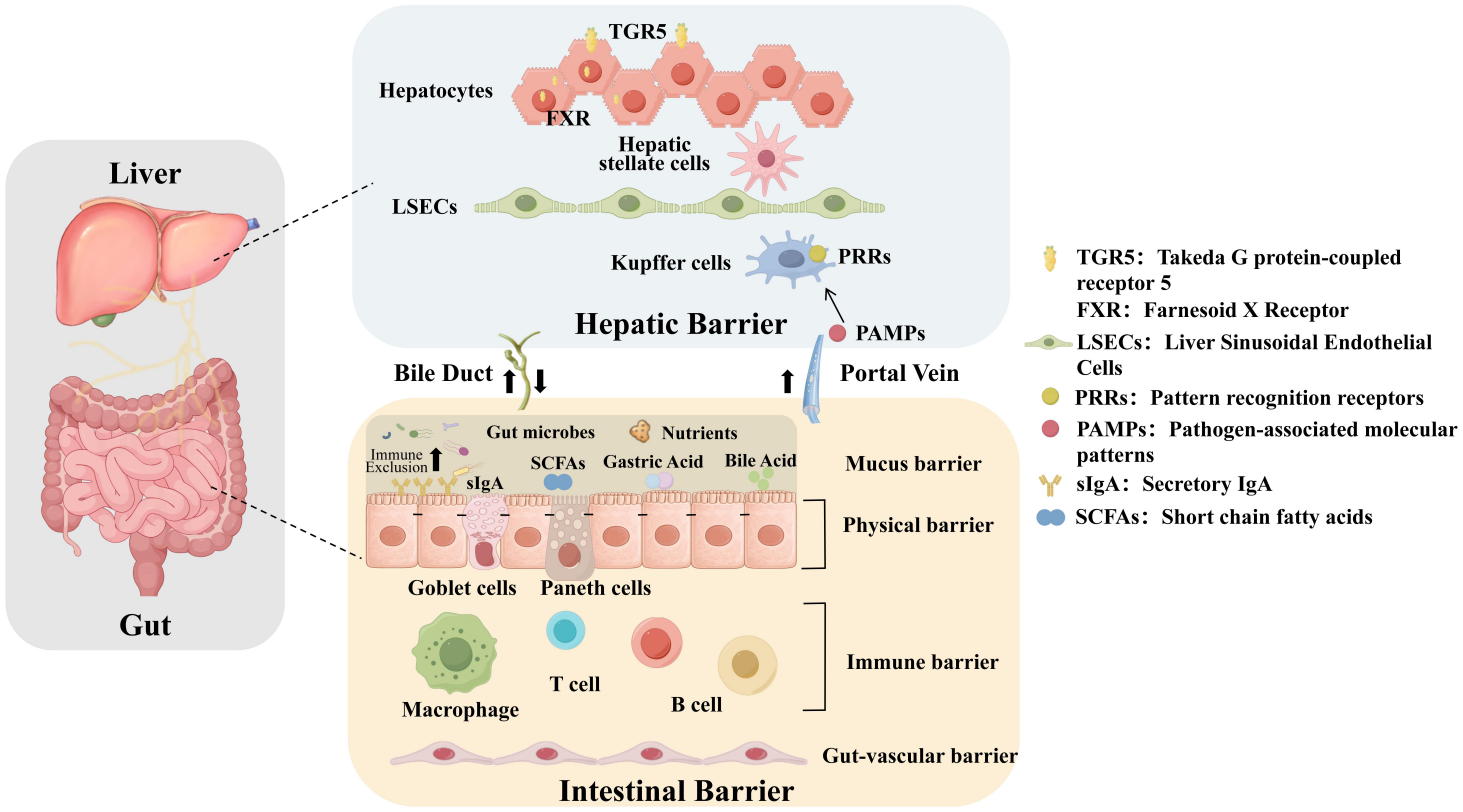
Schematic diagram of the gut-liver axis barriers and connections.

The gut-liver axis refers to the bidirectional regulatory system formed between the intestine and the liver through the portal venous circulation, enterohepatic circulation of bile acids, and exchange of immune factors. Approximately 70%−80% of the liver's blood supply comes from the portal vein, this unique circulatory system directly transports all nutrients absorbed by the intestines, microbial metabolites, and potential toxins through the portal vein first to the liver for metabolism and integration, and then redistributes them throughout the body (27, 28), which constitutes the physiological basis of the “gut-liver axis” (Figure 1). Its core regulatory mechanisms primarily involve the integrity of physical barriers, bile acids (BAs) circulate in the gut-liver, and the signal transduction of metabolic products.

### 2.1 The integrity of the physical barrier and intestinal permeability

Maintaining the normal function of the gut-liver axis is primarily dependent on the integrity of the intestinal mucosal barrier. This barrier is composed of a mechanical barrier (intestinal epithelial cells (IECs) and tight junctions (TJs)), a chemical barrier (mucous layer, antimicrobial peptides), an immune barrier, and a microbial barrier. TJs, such as Occludin, Claudin, and ZO-1, are crucial for controlling intestinal permeability (27, 29–33). When the gut microbiota is dysregulated, tight junctions are disrupted, leading to increased intestinal permeability. When the barrier is compromised, pathogen-associated molecular patterns (PAMPs), such as lipopolysaccharide (LPS), peptidoglycan, and bacterial DNA, as well as bacteria, enter the liver via the portal vein and bind to toll-like receptor 4(TLR4) on Kupffer cell surfaces, triggering myeloid differentiation primary response 88 (MyD88)-dependent signaling pathways, activating nuclear factor kappa-B(NF-κB) and AP-1 transcription factors, and inducing the release of pro-inflammatory cytokines, such as tumor necrosis factor-α (TNF-α), interleukin-1β (IL-1β), and interleukin (IL)-6, as well as chemokines, which are major triggers of liver inflammation and fibrosis (34–36). Meanwhile, these inflammatory responses disrupt lipid metabolism, elevating serum free fatty acid and triglyceride levels and promoting their accumulation in the liver (37).

### 2.2 BAs circulate in the gut-liver

The liver synthesizes primary bile acids from cholesterol and excretes them into the intestine with bile. In the intestine, gut microbiota convert them into secondary bile acids (such as deoxycholic acid, lithocholic acid) through the action of bile salt hydrolase (BSH) and 7α-dehydroxylase (38, 39). Bile acids are important signaling molecules, primarily activating two major classes of receptors. In the intestine, bile acids activate the farnesoid X receptor (FXR) in ileal epithelial cells, inducing the production of fibroblast growth factor 19 (FGF19). FGF19 enters the liver via the portal vein, binds to fibroblast growth factor receptor 4, and feedback-inhibits the expression of cholesterol 7α-hydroxylase, thereby controlling the rate of bile acid synthesis. Additionally, bile acids activate Takeda G protein-coupled receptor 5 (TGR5) on various cells (such as immune cells, enteroendocrine cells). TGR5 signaling regulates blood sugar by inducing the secretion of glucagon-like peptide-1 and has anti-inflammatory effects, protecting the intestinal barrier (40–43). It is noteworthy that there are significant differences in bile acid composition and the FXR/TGR5 signaling mechanism among various domestic animal and poultry species, and these differences directly affect the metabolic regulatory function of the gut-liver axis. In poultry, goose deoxycholic acid (CDCA) and its taurine-bound form (TCDCA) are the main components of bile acid pools (44, 45). Pigs have a unique bile acid composition, primarily consisting of porcine cholic acid (HCA) and porcine deoxycholic acid (HDCA). HCA promotes GLP-1 secretion through a unique mechanism that simultaneously activates TGR5 and inhibits FXR (46, 47). In contrast, the bile acid pool in ruminants is primarily composed of cholic acid (CA) and chenodeoxycholic acid (CDCA), and FXR signaling is involved in regulating postpartum energy metabolism and immune homeostasis (48). It suggests the species-specificity of the bile acid metabolism system and its impact on the regulation of gut-liver axis.

### 2.3 The signal transduction of microbial metabolites

The gut microbiota produces short-chain fatty acids (SCFAs) through the fermentation of dietary fiber and indole derivatives through the metabolism of tryptophan, profoundly influencing the metabolism of livestock and poultry hosts via the gut-liver axis (36, 49, 50). In terms of tryptophan metabolites, the gut microbiota converts tryptophan into derivatives such as indole-3-propionic acid (IPA), acting as ligands for the aryl hydrocarbon receptor to activate the IL-22 pathway, enhancing the integrity of the intestinal epithelial barrier and regulating liver immune homeostasis. Notably, in livestock and poultry, the composition and function of the gut microbiota have unique species-specific characteristics that significantly affect the regulation of the gut-liver axis. In ruminants, the rumen microbiota is dominated by several genera, including *R. flavefaciens, R. albus*, et al. (51, 52). The VFAs produced by rumen microbes after fermenting feed enter the liver through the portal vein, supporting gluconeogenesis and lipid metabolism (53, 54). In contrast, the gut microbiota of monogastric animals such as pigs and poultry primarily colonizes the hindgut (cecum/colon), with *Bacteroides, Lactobacillus*, and *Faecalibacterium* as the core genera. These genera produce butyrate, which constitutes a higher proportion of total SCFAs in monogastric animals compared to ruminants, reflecting their distinct digestive physiology and metabolic requirements (55).

## 3 The gut-liver axis in animal pathobiology

In the field of animal husbandry and veterinary medicine, particularly for intensive production of ruminants and pigs and poultry, research on the gut-liver axis holds extremely significant practical implications. In modern farming systems, animals often face multiple challenges such as high-concentrate feed diets, early weaning, and environmental stress, which often first disrupt gut tight junctions, leading to mild, persistent gut leakage (56–58). This low-grade inflammatory state can trigger a series of diseases, ultimately manifesting as visible economic losses such as reduced feed conversion efficiency and slower daily weight gain. Therefore, it is crucial to clarify the regulatory mechanisms of the gut-liver axis in animals. In this section, we summarize the key roles and regulatory mechanisms of the gut-liver axis in ruminants, pigs and poultry (Table 1).

**Table 1:** Core regulatory mechanisms of the gut-liver axis in different animals.

Animal categories	Related diseases	Core mechanisms	Prevention and control targets	References
Ruminants	Fatty liver, NASH-like metabolic disorders, postpartum metabolic diseases, hepatic abscesses, metabolic adaptation to plateau environments.	1. Energy disorders: Rumen microbial fermentation regulates hepatic energy metabolism via acetate-mediated AMPK-PPARα pathways. 2. Plateau adaptation: VFAs and microbial metabolites regulate hepatic gluconeogenesis-related enzymes (PC, PCK2) and transcription factors such as FOXO1, supporting metabolic adaptation. 3. Hepatic abscesses: Intestinal pathogens and endotoxins (e.g., Clostridium perfringens) enter the liver through portal circulation.	Regulation of rumen microbiota and fermentation patterns; nutritional modulation of VFA production; maintenance of intestinal barrier integrity; early control of pathogenic bacterial overgrowth.	(64, 65, 67–70, 132)
Swine	Hepatic inflammation, oxidative damage, fatty liver, cholestatic liver injury, fibrosis, metabolic disorders.	1. Barrier dysfunction and endotoxin translocation: Disruption of intestinal tight junctions increases permeability, allowing microbial endotoxins and metabolites to enter the liver. 2. Oxidative stress–inflammatory signaling: Crosstalk between Nrf2/Keap1 antioxidant signaling and TLR4/NF-κB inflammatory pathways mediates hepatic oxidative stress and inflammatory injury. 3. Abnormal regulation of lipid homeostasis by CAMKK2-AMPK pathway is involved in the pathogenesis of metabolic diseases. 4. Bile acid-mediated dysregulation of signaling pathways such as FXR and β-catenin regulates hepato-intestinal injury.	Enhancement of intestinal barrier function; modulation of oxidative stress (Nrf2 activation); regulation of bile acid metabolism and FXR signaling; microbiota-targeted nutritional interventions	(16, 71–76, 78–83)
Poultry	NAFLD, FLHS, exogenous contaminant-induced hepatic injury, lipid metabolism disorders.	1. Gut microbiota and their metabolites regulate hepatic fatty acid synthesis, bile acid circulation, and lipid deposition via pathways like PPAR. 2. Impaired intestinal barrier integrity, promoting translocation of gut-derived endotoxins into circulation, activating TLR4/MyD88/NF-κB signaling pathways. 3.Pollutant-amplified hepatotoxicity: Environmental contaminants disturb microbiota composition and bile acid metabolism, amplifying hepatic injury through the gut–liver axis.	Optimization of dietary structure; maintenance of gut microbiota stability; prevention of environmental contaminant exposure; nutritional strategies targeting inflammation and oxidative stress	(15, 21, 86–92)

### 3.1 Role of the gut-liver axis in ruminants

In ruminants, long-term feeding of a high-concentrate diet (HCD) is a common method to meet the energy demands for animal growth and production (dairy products, meat), which may lead to liver metabolic dysfunction (59, 60). Concurrently, the periparturient period represents a critical physiological window during which dairy cows and ewes experience marked appetite depression and reduced feed intake, leading to negative energy balance (NEB), or develop hepatic pathologies postpartum due to metabolic dysregulation—most notably hepatic ketosis and non-alcoholic fatty liver disease (NAFLD) (61–63). Emerging evidence has illuminated the pivotal role of gut microbiota alterations in the initiation and progression of ruminant liver diseases, thereby highlighting the central importance of the gut-liver axis in metabolic health (64). Specifically, Wang *et al*. demonstrated that abnormal proliferation of *Clostridium perfringens* can transmit pathological signals to the liver via the gut-liver axis, directly promoting fatty liver formation in dairy cows (65). Zhang et al. found that NAFLD caused by NEB is associated with a decrease in the abundance of *Ruminococcus spp*. in the ileum (66). Research has found that dysregulation of gut contents can inhibit acetic acid-mediated liver AMP-activated protein kinase-Peroxisome proliferators-activated receptor α (AMPK-PPARα) signaling pathway, thereby causing systemic energy metabolism disorders (67). For ruminants living in extreme environments, the gut-liver axis serves as a critical foundation for metabolic adaptation. Multi-omics analyses have revealed that plateau goats can maintain physiological homeostasis in high-altitude environments through the gut-liver axis (68). Yang *et al*. further elucidated that Tibetan sheep enhance their adaptation to hypoxic and low-temperature conditions through rumen microorganism–VFAs–VFAs transporter gene interactions on the key enzymes (pyruvate carboxylase, phosphoenolpyruvate carboxykinase) and gene, including forkhead box protein O1 (*FOXO1*) and mitochondrial phosphoenolpyruvate carboxykinase 2 (*PCK2*), related to gluconeogenesis (69). Additionally, studies have found that intestinal pathogens and their toxins can be transported via the gut-liver axis, inducing local inflammatory responses and, in severe cases, hepatic abscesses (70). In summary, the gut-liver axis plays a vital role in hepatic metabolic health, environmental adaptation, and disease pathogenesis in ruminants. This provides novel perspectives and approaches for preventing and treating related diseases through interventions targeting the gut-liver axis.

### 3.2 Role of the gut-liver axis in pigs

The gut-liver axis is a crucial integrated system for maintaining metabolic homeostasis and health in pigs. Through the bidirectional signaling network between the gut microbiota and the liver, it systematically regulates nutrient absorption, energy metabolism, immune defense, and detoxification functions (71–74). In modern commercial production, pigs face multiple stress challenges such as early weaning, rapid population changes, high-density housing, and extreme environments. These stressors disrupt the stability of the gut-liver axis, becoming a key pathological basis for the occurrence and development of diseases (16, 75). Research on the gut-liver axis based on pig models primarily focuses on the occurrence of various diseases under stress conditions, leading to reduced growth performance, with inflammation and barrier function being key areas of mechanistic study. At the molecular level, the interaction dysregulation between the Nrf2/Keap1 antioxidant pathway and the TLR4/NF-κB pro-inflammatory pathway constitutes the core mechanism of the enterohepatic axis mediating oxidative stress and liver inflammation in pigs. LPS released by intestinal microbiota dysregulation activated the liver TLR4/MyD88 axis, and was degraded by IκBα phosphorylation mediated by IKK, promoting NF-κB nuclear translocation and initiating pro-inflammatory gene transcription. At the same time, ROS modified the cysteine residue of Keap1 and relieved its inhibition of Nrf2. The key interaction was that NF-κB competitively binded to CBP/p300 to inhibit Nrf2 activity, while Nrf2-induced HO-1 could stabilize IκBα and negatively regulate NF-κB. Downstream NF-κB continued to activate and down-regulate the expression of intestinal occludin and claudin-1, increased intestinal permeability, and promoted more LPS to enter the liver, forming a vicious circle of “intestinal barrier destruction-liver inflammation-oxidative stress” (72, 76, 77). In addition to the oxidative stress-inflammatory pathway, the dysregulation of the production and transport of microbial metabolites plays a key role in maintaining the imbalance of the gut-liver axis. Metabolites such as secondary bile acids (e.g., ursodeoxycholic acid) and SCFAs (e.g., butyrate) regulate the balance of liver lipid synthesis and decomposition by activating nuclear receptors like FXR and PPAR. These metabolic regulations not only directly affect pig growth performance but are also closely related to the metabolic recovery of individuals with intrauterine growth restriction and the reallocation of energy adaptation under cold and heat stress (78–81). Among them, bile acids are the core mediators of gut-liver axis signaling. Targeting the FXR signaling pathway, secondary bile acid metabolic pathways, or related intracellular signals (such as β-catenin) can effectively alleviate cholestatic liver injury, liver fibrosis, and related intestinal inflammation (82, 83). Given the crucial role of the gut-liver axis in maintaining pig health, nutritional intervention and optimizing gut-liver axis function have become important directions in modern pig nutrition research.

### 3.3 Role of the gut-liver axis in poultry

Environmental and nutritional factors, such as light cycles, dietary composition and differences in rearing system, directly affect bile acid metabolism or the composition and activity of gut microbes via this axis, thereby altering hepatic lipid metabolism, as well as the nutrient utilization efficiency and production performance of poultry (84, 85). This process involves multiple molecular mechanisms. The gut microbiota produces secondary bile acids, SCFAs and tryptophan metabolites as key messengers, which circulate through the portal vein to the liver, activate nuclear receptors such as PPARs, and regulate fatty acid β-oxidation, bile acid circulation, and lipid synthesis and deposition (15, 86, 87). For example, under a high-energy and low-protein diet, the expression of PPARα in the liver is down-regulated, resulting in inhibited fatty acid β-oxidation. At the same time, carbohydrate response element-binding protein-1c (SREBP-1c) is activated, which in turn drives the synthesis of triglycerides and induces FLHS, which can be mitigated by optimizing dietary structure (77, 88). However, when the gut-liver axis homeostasis is disrupted, the hepatotoxic effects of exogenous stressors and pollutants (such as microplastics, heavy metals, pesticide residues) will be significantly amplified (89–91). The core mechanism is that intestinal dysbiosis leads to abnormal metabolism, while intestinal barrier function is compromised, LPS enters the bloodstream, activating the liver's TLR4/MyD88/NF-κB inflammatory pathway, triggering a cascade of inflammatory reactions and liver cell damage (90, 92, 93).

## 4 Therapeutic potentials and interventions

The gut-liver axis plays a critical role in regulating metabolism, immune function, and barrier integrity in livestock and poultry. Emerging strategies—including dietary interventions (such as SCFAs and amino acids), probiotics, prebiotics, and fecal microbiota transplantation—demonstrate promising potential for modulating this axis to improve liver health and overall metabolic status (Table 2).

**Table 2:** Clinical applications of the gut-liver axis.

Interventions	Strategy	Application model	Core effects	Application limitations	References
Dietary intervention	β-hydroxy-β-methylbutyrate (HMB)	Broilers	0.10% dietary supplementation modulates gut microbiota and reduces hepatic fat deposition.	Effects may depend on diet composition and growth stage.	(94)
	L-arginine/N-carbamylglutamate	Suckling lambs (IUGR)	1% supplementation improves colonic barrier function, increases SCFA production, and alleviates oxidative stress and inflammation.	Response varies with developmental stage and metabolic status.	(95)
	Sodium butyrate (encapsulated)	Laying hens	250–1,000 mg/kg supplementation reduces hepatic lipid accumulation and alleviates FLHS.	Stability and release efficiency of coated butyrate affect efficacy.	(97)
	β-hydroxybutyrate (BHB)	Piglets (LPS model)	25 mg/kg/day supplementation alleviates hepatic inflammation and improves energy metabolism and growth performance.	Effects primarily verified under experimental stress models.	(98)
Probiotics/prebiotics	*Lactobacillus paracasei* (microencapsulation)	Broilers	Reduces systemic oxidative stress, alleviates hepatic metabolic burden.	Strain-specific effects; stability during feed processing required.	(104)
	*Bacillus licheniformis*	Laying hens	Increases hepatic antioxidant enzyme activity (e.g., GSH-Px, SOD).	Dose optimization and host–strain compatibility required.	(107)
	*Bacillus subtilis*	Broilers (heat stress model)	Improves intestinal morphology, enhances antioxidant capacity, and reduces hepatic HSP70 expression under stress	Effects influenced by environmental conditions and strain variation.	(110, 111)
Microbiota transplantation	FMT (single application)	Broilers, weaned piglets, calves	Improves growth performance, alleviates diarrhea, enhances intestinal barrier integrity.	Donor screening, pathogen transmission risk, unstable microbial colonization.	(118, 133)
	FMT + inulin	Chicks	Enhances intestinal barrier function and promotes early immune development of GALT through CD28/CTLA4 signaling.	Standardization of donor microbiota and prebiotic dosage required.	(119)
Virome-based therapy	Fecal virome transplantation(FVT)	Broilers (LPS inflammation model)	Restores tight-junction protein expression, reduces inflammatory responses, improves nutrient absorption without introducing live bacteria.	Virome composition complexity and lack of standardized preparation protocols.	(124, 125)

### 4.1 Dietary intervention

In livestock health farming, the direct addition of functional nutrients through the diet has become a core strategy for precise regulation of the gut-liver axis and improvement of metabolic health. Current research primarily focuses on the regulatory effects of amino acids and SCFAs on host health via the gut-liver axis. For instance, Under normal healthy conditions, adding 0.10% of β-hydroxyβ-methylbutyrate (HMB) to feed can regulate the gut microbiota of meat chickens and inhibit liver fat deposition (94). Under pathological conditions, dietary intervention targeting the gut-liver axis appears particularly important. In an intrauterine growth retardation (IUGR) lamb model during the suckling period (21–28 days after birth), dietary 1% L-arginine or 0.1% N-Carbamylglutamate supplementation not only significantly enhanced colonic barrier function but also alleviated oxidative stress and inflammatory responses by modulating gut microbiota and promoting SCFAs production (95). A study by Zheng *et al*. shown that butyrate can alleviate hepatic steatosis induced by a high-fat low-fiber diet by activating the hepatic GPR41/43–CaMKII/HDAC1–CREB pathway (96). Miao *et al*. supported this mechanism through farming practices, feeding a basal diet supplemented with 0, 250, 500, 750 and 1,000 mg/kg coated sodium butyrate for 8 weeks significantly reduces liver fat content in laying hens and alleviates FLHS (97). Importantly, this effect is not limited to a single animal species or health status. In an LPS-induced stressed piglet model, dietary supplementation of β-hydroxybutyrate (BHB, a metabolite of butyrate) (25 mg/kg/days) alleviated liver injury and inflammation by modulating glucose and lipid metabolism, improved energy metabolism, and consequently enhanced growth performance (98). In addition to butyrate, dietary propionate supplementation can modulate the gut microbiota in broilers to inhibit fat deposition (99). In addition to directly supplementing SCFAs or their precursors, natural plant feed additives and extracts can also exert protective effects through the gut-liver axis (100, 101). For example, flaxseed powder can regulate gut microbiota and bile acid metabolism through the gut-liver axis, alleviating non-alcoholic fatty hepatitis (102). This discovery expands the range of materials available for nutritional intervention using the gut-liver axis. In summary, the strategy of supplementing specific amino acids and nutrients to protect liver health through the gut-liver axis has broad applicability, and in the future, attention should continue to be paid to the differences in responses to nutritional interventions by the gut-liver axis at different physiological stages and genetic backgrounds, as well as the interaction between functional nutrients and gut microbiota. Notably, the regulatory efficacy of dietary interventions targeting the gut–liver axis can also be influenced by environmental conditions in production systems. Factors such as heat stress and stocking density may alter gut microbiota composition, intestinal barrier integrity, and systemic inflammatory status, thereby modifying host metabolic responses to nutritional strategies (103).

### 4.2 Probiotics and prebiotics

Research has confirmed that probiotics or prebiotics can improve livestock health and metabolic status through multiple pathways mediated by the gut-liver axis. Under healthy conditions, they can regulate the antioxidant capacity of the liver. For example, Gyawali *et al*. found that targeted delivery of *Lactobacillus paracasei* to the intestines of broilers via microencapsulation technology can optimize gut microbiota structure and enhance intestinal barrier function, thereby systemically reducing oxidative stress and alleviating hepatic metabolic burden (104). *Lactobacillus* improves the growth performance of weaned piglets by upregulating the expression of intestinal amino acid transporters to promote amino acid transport via the gut-liver axis, while simultaneously inhibiting the synthesis of extracellular matrix (ECM) components (105), *Lactobacillus delbrueckii* induces modifications in the ileal microbiota, enhancing the deconjugation and excretion of bile acids in developing pigs (106). Similarly, Pan *et al*. reported that feeding *Bacillus licheniformis* can increase the concentrations of glutathione peroxidase (GSH-Px) and superoxide dismutase (SOD) in the liver (107). An intact intestinal barrier helps prevent the translocation of pathogenic microorganisms (34). For example, dietary resveratrol supplementation can repair intestinal structure, modulate microbial balance, and enhance the expression of genes related to the intestinal barrier (108). Meanwhile, Wang *et al*. observed that dietary supplementation with *Bacillus subtilis* can mitigate heat stress-induced behavioral and inflammatory responses through microbiota-mediated immune regulation, thereby enhancing the host's ability to cope with stress (109). Interestingly, beyond direct probiotic or prebiotic supplementation, probiotic preparations derived from *Bacillus subtilis* can not only improve intestinal health through conventional pathways (e.g., enhancing intestinal morphology, optimizing microbial community composition, and increasing antioxidant enzyme activities, including GSH-Px and SOD), but also downregulate the expression of heat shock protein 70 (HSP70) in hepatocytes under heat stress, thereby further strengthening intestinal health (110, 111). Therefore, even under stressful conditions, probiotics can restore and reinforce gut-liver axis function by repairing the intestinal barrier and regulating immunity and inflammation, thus systematically correcting metabolic disorders. However, the effect of probiotics on improving the health of livestock and poultry through the gut-liver axis has significant strain-host specificity (112, 113), in practical applications, the stability of probiotics under the influence of various factors should be fully considered (114), while active standards after processing should be established and strains developed for different types of animal feed (115).

### 4.3 Fecal microbiota transplantation (FMT)

In recent years, fueled by growing research interest in the gut microbiota, FMT has emerged as a prominent field of investigation. In human medicine, FMT has demonstrated significant clinical value (116). The FMT process involves transplanting the functional microbiota from a healthy donor's gut to a recipient to help reconstruct their normal microbiota, thereby achieving disease resistance and health maintenance (117). Liu *et al*. demonstrated that early-life FMT promotes broilers growth by maintaining *Lactobacillus* abundance in the jejunum and activating the hepatic growth hormone/insulin-like growth factor-1 (GH/IGF-1) signaling pathway (118). Notably, the effects of FMT are not singular, and synergistic combinations with prebiotics and other substances are demonstrating enhanced regulatory potential. For instance, Song *et al*. confirmed that the combined application of FMT and inulin significantly promotes the early immune development of gut-associated lymphoid tissue (GALT) in chicks by upregulating key immune genes, enhancing intestinal barrier function, and identified the Cluster of differentiation 28/Cytotoxic T-Lymphocyte-Associated Antigen 4 (CD28/CTLA4) signaling axis as a crucial regulatory mechanism. This suggests that synergistic strategies can further optimize and directionally enhance the immune-promoting functions of FMT, providing new insights for healthy breeding practices (119). However, FMT faces challenges in transitioning from laboratory research to large-scale industrial application, including difficulties in donor standardization, risks of pathogen transmission, and unstable microbial colonization (120, 121). Therefore, there are limitations in its application to livestock and poultry production, requiring strict screening of donors to ensure the safety of the donor gut microbiota.

### 4.4 Fecal virome transplantation (FVT)

Fecal virome transplantation (FVT) has recently emerged as a novel microbial intervention strategy distinct from conventional FMT (122). Unlike FMT, which transfers the entire microbial community including live bacteria, FVT primarily transfers viral components—particularly bacteriophages—thereby reshaping the gut microbiota in a more targeted and potentially safer manner (123). Interestingly, Feng *et al*. can induce potential adverse effects in chickens, such as decreased jejunal villus height and increased crypt depth. In contrast, fecal virome transplantation (FVT), a newer intervention strategy, reshapes the gut microbiota in a gentler manner and significantly enhances intestinal barrier function and nutrient absorption capacity. Furthermore, Wu *et al*. reported that in a LPS-induced acute intestinal inflammation model, FMT not only failed to effectively suppress the expression of pro-inflammatory cytokines and the enrichment of virulence-related genes but also potentially exacerbated intestinal barrier dysfunction and disrupted host metabolic homeostasis. In comparison, FVT significantly downregulated inflammatory responses, maintained secretory IgA levels, inhibited the spread of pathogenic genes, and effectively restored tight junction protein expression. This demonstrates that FVT can achieve safer and more efficient intestinal repair without introducing exogenous live bacteria, highlighting its unique potential as a novel microbial intervention strategy (124, 125). These findings indicate that FVT can achieve intestinal repair without introducing exogenous live bacteria, highlighting its advantages in safety and immune modulation (126). Compared with FMT, FVT may therefore represent a more controllable and biologically precise strategy for microbial ecosystem regulation, particularly under inflammatory or pathogen-challenged conditions.

## 5 Conclusion and future directions

This review systematically describes the gut-liver axis as a core regulatory network integrating the gut microbiota and liver metabolic function, and its hub role in maintaining the health of ruminants, pigs, and poultry. The pathogenic mechanisms shared in different animals are mainly manifested by intestinal barrier disruption, leading to changes in microbial metabolites and translocation, which then triggers a series of diseases including liver inflammation and lipid deposition. On this basis, we emphasize the importance of BA receptors and SCFAs as hub regulators in different animals, which are the theoretical basis for a wide-ranging nutritional intervention. At the same time, we also compare the unique microbial environments of ruminants, pigs, and poultry, providing a key basis for understanding species-specific intervention strategies.

In the future, to address challenges such as antibiotic resistance, high implementation costs, and environmental pressures, multi-omics approaches should be employed for in-depth research on the gut-liver axis to identify key species-specific biomarkers and core regulatory nodes (127, 128). For example, for metabolic syndrome during the peripartum period in ruminants, combining rumen metagenomics, serum metabolomics, and liver proteomics can identify early warning biomarkers to design precision nutritional formulations. Advanced technologies such as artificial intelligence (AI) and big data modeling should be utilized to integrate multi-dimensional data, including feeding records, physiological indicators, and omics data, to develop personalized nutrition and regulatory solutions (129, 130). Additionally, future research should focus on the interactions between the gut-liver axis and other organ axes (such as the gut-liver-brain axis, gut-liver-ovary axis, gut-liver-kidney axis, and gut-liver-muscle axis), developing interventions that actively regulate multiple organ axes simultaneously, and exploring new strategies for multi-axis synergistic regulation for livestock health management (131). These efforts will provide essential technical support for the sustainable development of the livestock industry and the improvement of animal welfare.
